# Stabilized Wide Bandgap MAPbBr*_x_*I_3–*x*_ Perovskite by Enhanced Grain Size and Improved Crystallinity

**DOI:** 10.1002/advs.201500301

**Published:** 2015-12-07

**Authors:** Miao Hu, Cheng Bi, Yongbo Yuan, Yang Bai, Jinsong Huang

**Affiliations:** ^1^ Department of Mechanical and Materials EngineeringUniversity of Nebraska–LincolnLincolnNebraska68588USA

**Keywords:** crystallinity, light stability, perovskite, tandem, wide bandgap

## Abstract

**The light instability of CH_3_NH_3_PbI*_x_*Br_3–*x*_ is one of the biggest challenges** for its application in tandem solar cells. Here we show that an improved crystallinity and grain size of CH_3_NH_3_PbI*_x_*Br_3–*x*_ films could stabilize these materials under one sun illumination, improving both the efficiency and stability of the wide‐bandgap perovskite solar cells.

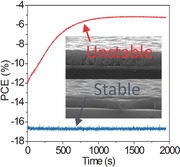

Methylammonium lead trihalide perovskite (MAPbX_3_, where MA is methylammonium, and X is a halide)‐based solar cells have been intensively investigated recently,^1^ with the demonstrated certified solar power conversion efficiency (PCE) exceeding 20%.^2^ To further boost the PCE to beyond the Schockley–Queisser limit,^3^ tandem structured solar cells have been investigated based on integrating MAPbX_3_ and low bandgap solar cells.^4^, ^5^, ^6^, ^7^, ^8^ However, the efficiency of the two‐terminal integrated perovskite‐silicon tandem cells is still low. The best reported efficiency of 13.7% for this type of tandem cells is far smaller than the individual cells yet, partially due to the limited performance of the mixed‐halide perovskite MAPbBr*_x_*I_3–*x*_ solar cell employed in this structure.^5^ The mixed‐halide perovskite MAPbBr*_x_*I_3–*x*_ is still one of the most promising candidates as the wide‐bandgap light absorber for the tandem application to match the bandgap of silicon, considering its continuously tunable bandgap from 1.6 eV to 2.3 eV with different bromide incorporation ratio.^7^, ^9^, ^10^ However, the application of MAPbBr*_x_*I_3–*x*_ based solar cells has been reported to confront with one big challenge of intrinsic light instability.^11^ The MAPbBr*_x_*I_3–*x*_ materials on mesoporous scaffold were shown to be unstable under illumination with a photo‐excited phase‐separation into two phases, one iodine‐rich phase and one iodine‐poor phase.^11^, ^12^ The lower bandgap phase thus acts as the charge traps, which was hypothesized to be responsible to the severely reduced device open circuit voltage and device PCE for the mixed‐halide perovskite devices.^6^, ^13^, ^14^

In this manuscript, we report that the mixed‐halide perovskite, MAPbBr*_x_*I_3–*x*_—with an optical bandgap of 1.70–1.75 eV—are stable under illumination with the improved film microstructures. The application of a non‐wetting hole transporting layer was found to increase the grain size dramatically and stabilize the MAPbBr*_x_*I_3–*x*_ grains, which also improved the PCE of the wide bandgap MAPbBr*_x_*I_3–*x*_ perovskite devices to 16.6%, the highest reported value for wide bandgap perovskite solar cells.

It has been calculated that a bandgap between 1.70–1.76 eV is optimal for the top cell to get a PCE of >30% in the tandem devices with c‐Si in the detailed balance study, which was targeted in this study.^15^ Interdiffusion method was applied to fabricate phase homogeneous, pin‐hole free MAPbBr*_x_*I_3–*x*_ thin films by the reaction of PbI_2_ with MAI*_y_*:MABr_1–*y*_ mixed‐halide organic precursors. The bandgap was tuned by controlling the MABr percentage in the MAI*_y_*:MABr_1–*y*_ organic precursors.^16^, ^17^ The best PCE achieved with the interdiffusion formed MAPbBr_0.6_I_1.4_ planar‐heterojunction structure solar cells was 13.1%, in which the hole transport layer was poly(3,4‐ethylenedioxythiophene) polystyrene sulfonate (PEDOT:PSS).^10^ In this work, we further increase the efficiency of the device based on the similar perovskite composition to 16.6% by employing poly[bis(4‐phenyl)(2,4,6‐trimethylphenyl)amine] (PTAA) as the hole transport layer, which has a non‐wettability to the perovskite precursors.^18^

**Figure** **1**a shows the current density (*J*)–voltage (*V*) curves of the optimized MAPbBr*_x_*I_3–*x*_ cells with a planar‐heterojunction structure of ITO/PTAA/MAPbBr*_x_*I_3–*x*_/[6,6]‐phenyl C_61_‐butyric acid methyl ester (PCBM)/C_60_/9‐dimethyl‐4,7‐diphenyl‐1,10‐phenanthroline (BCP)/Al,^16^, ^19^, ^20^ in which the device performance parameters for MAPbBr_0.5_I_2.5_ (black curves) can be derived as a *J*_sc_ of 18.3 mA cm^−2^, an open circuit voltage (*V*_oc_) of 1.16 V, a fill factor (*FF*) of 78.2%, and a PCE = 16.6%. We further increased Br concentration in methylammonium halide precursor up to 64% (or a MAI:MABr weight ratio of 1:1.3)—the optical bandgap of MAPbBr*_x_*I_3–*x*_ was increased to 1.75 eV. We assign the perovskite with this composition as MAPbBr_0.8_I_2.2_ based on the lattice parameter derived from the XRD pattern. The device parameters for this wider‐bandgap MAPbBr_0.8_I_2.2_ extracted from the red curves in Figure 1a are *V*_oc_ = 1.21 V, *J*_sc_ = 15.8 mA cm^−2^, *FF* = 77.9%, and PCE = 14.9%.

**Figure 1:**
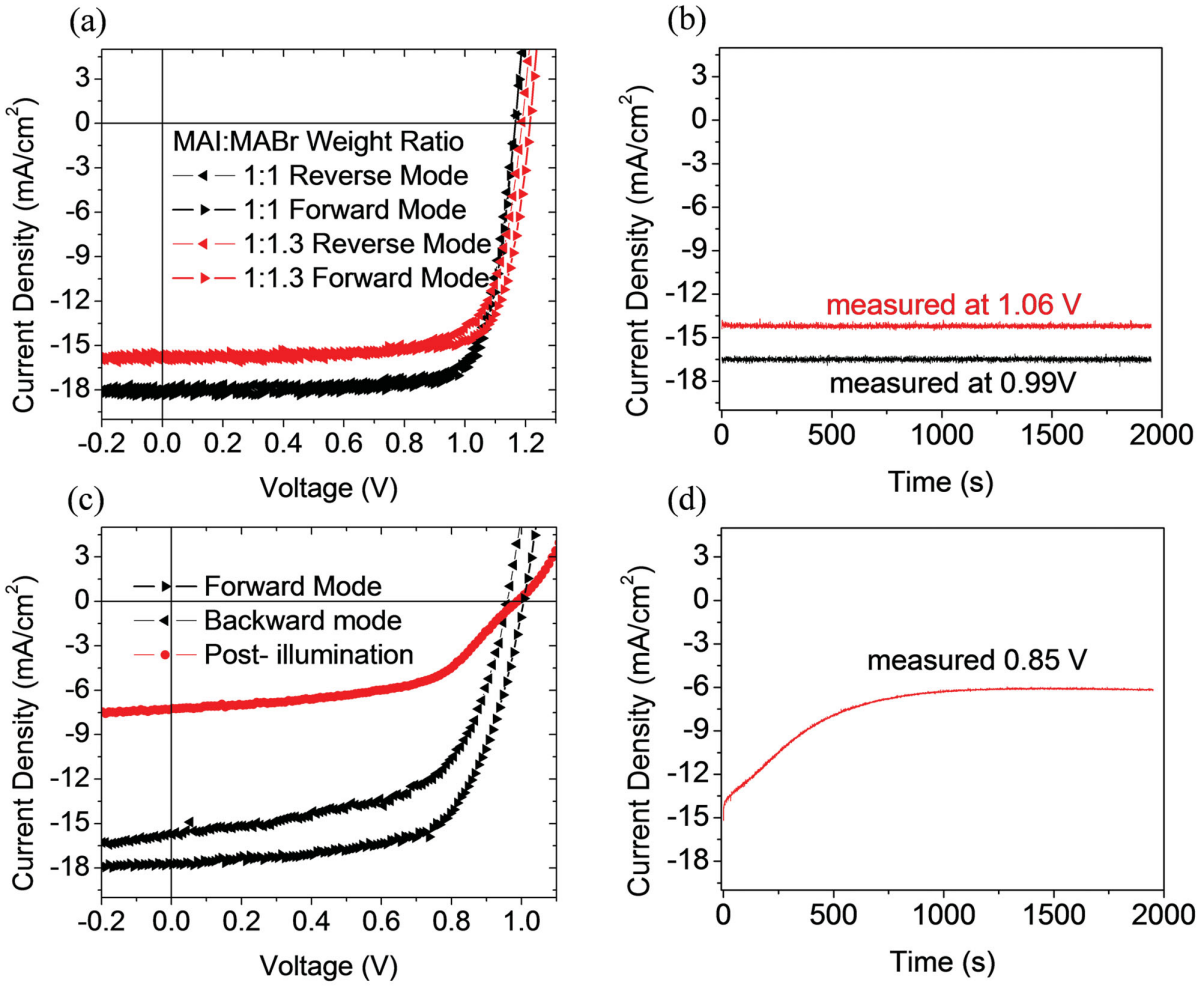
a,c) *J–V* curves for the optimized MAPbBr*_x_*I_1–*x*_ cells fabricated on PTAA on a)PEDOT:PSS, c) hole transport layers, measured with a rate of 0.6 V min^−1^ in increasing (from *J*_sc_ to *V*_oc_) and decreasing (from *V*_oc_ to *J*_sc_) bias under simulated AM 1.5G illumination. b,d) Photocurrent density measured at the maximum power output point for the devices with the MAPbBr*_x_*I_1–*x*_ grown on b) PTAA, d) PEDOT:PSS. The bias for the photocurrent measurement is labeled in the figures.

In addition to the higher PCE achieved in this study, the wide bandgap MAPbBr*_x_*I_3–*x*_ devices studied in this work also showed improved photo‐stability, in striking contrast to what was reported previously.^9^, ^11^, ^12^ The steady‐state photocurrent output at the maximum power output point of the cells are shown in Figure 1b and the applied bias was labeled, which shows almost constant output over 30 min testing under one sun illumination. The steady‐state photocurrents also directly confirmed the steady‐state PCEs, or stabilized PCE, which consists with those derived from the *J–V* curves. In order to find out the mechanism for the improved solar cell PCE and photo‐stability for these devices, we fabricated the control cells with PEDOT:PSS as the hole transport layer and all other layers fabricated with exactly the same protocol. It should be noted that no solvent annealing was applied, which is different from our previous study,^21^ because the perovskite films on top of PTAA do not need a solvent annealing process to form large grains. The typical *J–V* curves for these structured devices is shown Figure 1c which showed a smaller *FF* and larger photocurrent‐hysteresis when the scanning direction was flipped. As shown in Figure 1d, the photocurrent at the maximum‐power output point dropped with illumination time rapidly at first ten minutes, which explains the large hysteresis for this device. Then the photocurrent output saturated at –6 mA cm^−2^, which agrees with the *J–V* curve collected after the light‐stability test (red curve in Figure 1c). The maximum power output reduced from 14.9% to 3.9% after illumination at one sun for 1000 s. Since the cells in Figure 1a and Figure 1b were fabricated with the same composition perovskite and procedures, the photovoltaic performance difference must be caused by the different hole transport layers applied.

PEDOT:PSS has been used in planar heterojunction structured perovskite solar cells at the initial stage of our research;^16^, ^19^ however, the *V*_oc_ of the devices with PEDOT:PSS HTL is generally smaller than best reported devices. PTAA was employed in this work with the initial intention of reducing the *V*_oc_ loss due to its lower HOMO than that of PEDOT:PSS.^13^, ^22^ We did observed increase of the *V*_oc_ to 1.17 V from 1.01 V for MAPbBr_0.5_I_2.5_ devices when replacing PEDOT:PSS with PTAA. The *V*_oc_ increase of 0.16 V is larger than the work function difference of 0.04 eV between PTAA and PEDOT:PSS measured by KPFM, indicating additional contribution from reduced charge recombination.^10^ We ascribe the morphology change of the perovskite films to the increased *V*_oc_ and improved device stability.

In order to find the origin of the improved photo‐stability of MAPbBr*_x_*I_3–*x*_, we studied the morphology difference of the MAPbBr_0.8_I_2.2_ films formed on PTAA and PEDOT:PSS hole transport layers. We first examined MAPbBr_0.8_I_2.2_ films formed on PTAA and PEDOT:PSS formed by the exactly same process. Both films have comparable thickness of around 350 nm. As shown by cross‐section scanning electron microscopies (SEM) of the films in **Figure** **2**a–b, the MAPbBr_0.8_I_2.2_ grains formed on PTAA are much large than those formed on PEDOT:PSS. The lateral size of the grains grown on PTAA are several times of the film thickness, while the grains grown on PEDOT:PSS are much smaller than the film thickness. It should be noted that the MAPbBr_0.8_I_2.2_ films formed here only went through the thermal annealing, rather than the solvent annealing, therefore the MAPbBr_0.8_I_2.2_ films on PEDOT:PSS have much smaller grains than what previously reported.^10^ The formation of the large grains on PTAA can be explained by the hydrophobic nature of PTAA surface because it affects the nucleation and grain growth behavior.^18^ The first step of organic–inorganic trihalide perovskite (OTP) film formation is OTP nucleation on the substrates after the chemical reaction of PbI_2_ and MAX. A wetting surface to OTPs, such as PEDOT:PSS, with small contact angle (*θ*) reduces the Gibbs free energy barrier for nucleation (Δ*G*_het_) by a factor that determines by the contacting angle:

(1) $$\Delta G_{\mathrm{het}}=\frac{\Delta G_{\mathrm{hom}(2+\cos\theta )(1-\cos\theta )}^{2}}{4}$$

(2) $$C^{*}=C_{0}\exp\left(-\frac{\Delta G_{\mathrm{het}}}{kT}\right)$$

where Δ*G*_hom_ is homogeneous nucleation energy barrier, *C*_0_ is the number of atoms per unit volume in the phase; C* is the concentration of critical sized nuclei; *k* is Boltzmann constant; *T* is temperature. For instance, a small *θ* of 10° reduces Δ*G*_het_ to be 1.7 × 10^−4^ Δ*G*_hom_, which dramatically promotes the nucleation and forms very dense nuclei on the wetting surface. While on a nonwetting surface where *θ* approaches 180°, the Δ*G*_het_ is comparable to Δ*G*_hom_, which suppresses nucleation and results in larger spacing between nucleuses and thus the formation of larger grains by the end of the initial stage of film drying. The followed thermal annealing induces the OTP grain growth. The formation of small grains on non‐wetting surface is not favored from energy point of view, and the grain boundaries tend to be in the out of plane direction to minimize the total grain boundary area. This mechanism is illustrated in Figure 2a,b together with the corresponding cross‐section scanning electron microscope (SEM) images of the films. Interestingly, a new phenomenon we observed is that when the MAPbBr_0.8_I_2.2_ perovskite film is as thick as 540 nm, the resulting MAPbBr_0.8_I_2.2_ films have reduced crystallinity and smaller grains in the region closer to the bottom of the films, but still large grains formed on the upper level. This may be explained by the fact that the substrate surface energy loses its influence on the grain formation at the upper level area when the film is too thick.^23^ The nucleation process near the bottom level could get greatly promoted by the fast cooling rate caused by the much cooler substrate than the solution, which results in denser nuclei and smaller grain size. Therefore, not only the substrate surface energy but also the thickness of the thin film can influence the microstructure of perovskite thin films.

**Figure 2:**
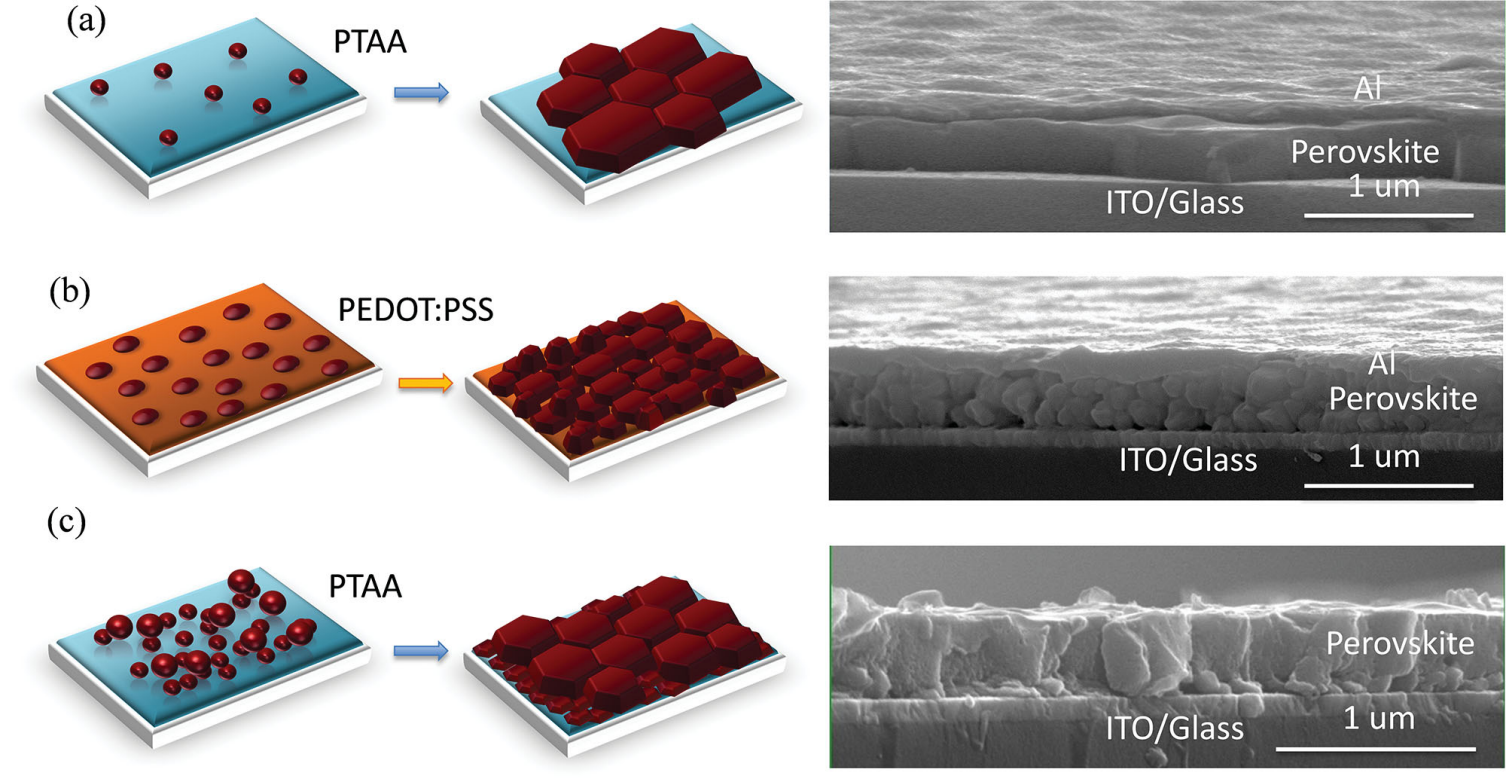
Schematic illustration of nucleation and growth of grains on wetting and non‐wetting hole transport layer surface and the corresponding cross‐section SEM images, for 328 nm thick MAPbBr_0.8_I_2.2_ thin film grown on a) PTAA, b) 361 nm thick MAPbBr_0.8_I_2.2_ thin film grown on PEDOT:PSS, c) 540 nm thick MAPbBr_0.8_I_2.2_ thin film grown on PTAA.

Another piece of evidence was found to support the microstructure of MAPbBr_0.8_I_2.2_ determines its photo‐stability by comparing the MAPbBr_0.8_I_2.2_ films with different thickness on PTAA HTL. As shown in **Figure** **3**a, the device with a 320 nm MAPbBr_0.8_I_2.2_ film, which has large and crystalline grains, was stable under one sun illumination for 33 min, while the devices with thicker MAPbBr_0.8_I_2.2_ active layer showed quickly degraded photocurrent under illumination. The optical bandgaps of the MAPbBr_0.8_I_2.2_ films with different thicknesses are the same, as shown by the absorption spectra in Figure 3b, which excluded the contribution from the different composition to the different degradation behavior of these films. This can be explained by that fact that excess organic precursor is always applied to deplete PbI_2_ in interdiffusion methods.

**Figure 3:**
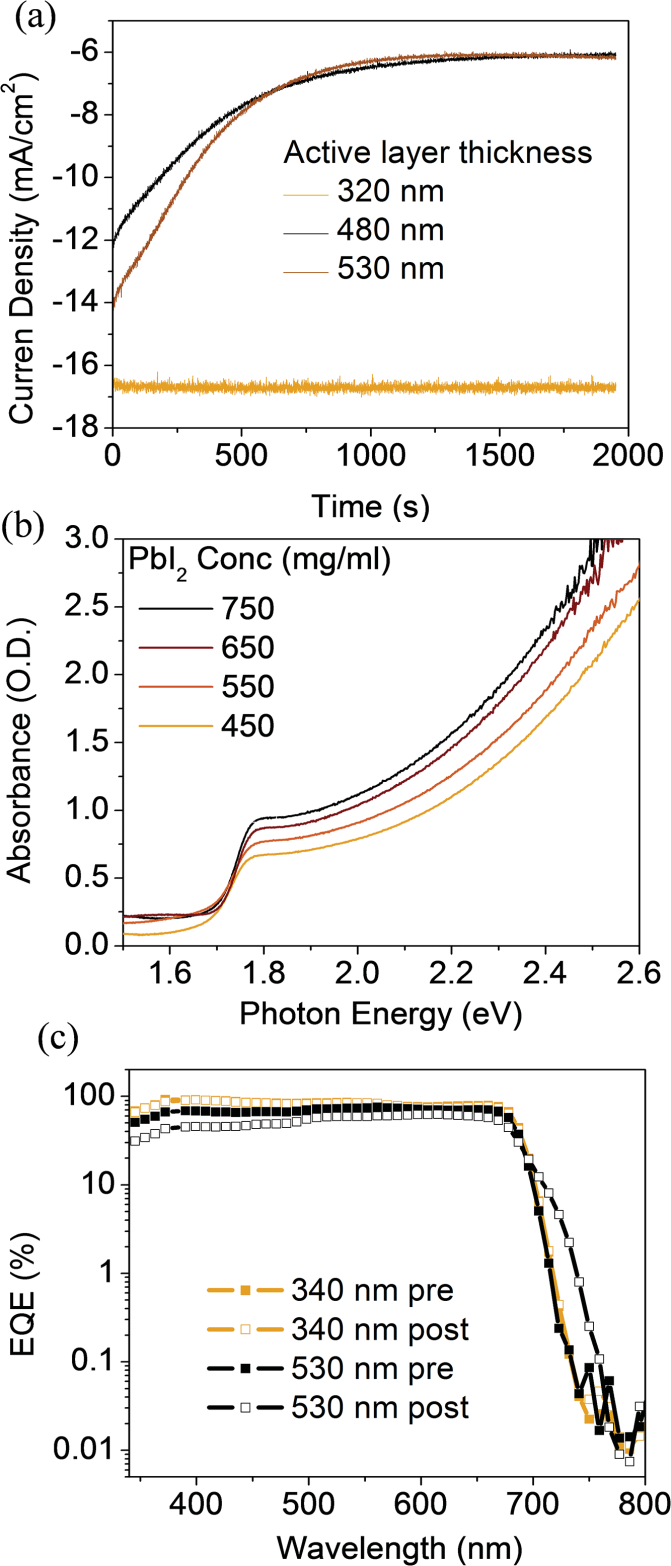
a) Photocurrent measured at the maximum power output point for the three devices with different thickness exposed to the simulated AM 1.5 G illumination for 33 min. b) Absorption spectra of the MAPbBr_0.8_I_2.2_ films with different thickness, which was controlled by the PbI_2_ precursor concentration. c) EQE spectra before (square) and after (circle) illumination under simulated AM 1.5 G for 20 min for the MAPbBr_0.8_I_2.2_ cells with 320 nm (orange) and 540 nm (black) MAPbBr_0.8_I_2.2_ film layers.

Further optical and material structure study confirmed the influence of microstructure on the grain photo‐stability. The photo‐induced phase separation generates a low‐bandgap phase which should cause photocurrent contribution from this new phase. Figure 3c shows the external quatum efficciency (EQE) spectra of the stable cell and unstable one before and after exposed to illumination. As expected, there is no change of EQE from the stable device with 320 nm active layer. It should be noted that the EQE spectra were plotted with logarithm operation. The highest EQE value is 90.5% from the device with 340 nm thick active layer at the wavelength of 374 nm. For the unstable device with 540 nm MAPbBr_0.8_I_2.2,_ the post‐illumination EQE reduced significantly for above bandgap excitation, while increased for below bandgap excitation (720–750 nm), which can be assigned to the photocurrent contribution from the new low bandgap phase. The very low EQE from this new phase indicates the strong trapping effect of it. Another notable finding is the photocurrent dropped more at the shorter wavelength (350 nm to 550 nm) range, which indicates the phase separation predominately occurs in the film region close to the PTAA side.

To increase the confidence of the conclusion, we investigated the light stability on the nude MAPbBr_0.8_I_2.2_ thin films with X‐ray diffraction (XRD) measurement and photoluminescence (PL) measurement, which were previously employed to identify the phase separation in this type of materials.^9^, ^11^, ^12^, ^24^ **Figure** **4**a compares the XRD pattern with normalized intensity for the stable MAPbBr_0.8_I_2.2_ film (320 nm) and the unstable one (530 nm) before exposing them to strong illumination. Although only a small amount of bromide was introduced, a cubic *Pm‐3m* group is identified in the MAPbBr_0.8_I_2.2_ XRD pattern with the characteristic peaks of (100), (110), (111), (200), (210). The peak positions are identical, which agrees with our earlier claim that the composition of these films does not change with the MAPbBr_0.8_I_2.2_ polycrystalline microstructure. However, the increased (100) peak intensity compared to other peaks for the stable MAPbBr_0.8_I_2.2_ suggests that the thinner MAPbBr_0.8_I_2.2_ polycrystalline film is better oriented than the thicker one, which gives new microstructure difference between these two compositional identical thin films in addition to the crystallinity and grain size. The random oriented MAPbBr_0.6_I_2.4_ generally contains larger‐angle grain boundaries, which would enhance the halide migration to assist the phase segregation. By looking at the (200) peak pre‐ and post‐illumination of the two films (Figure 4b,c), we clearly observed a peak splitting from the unstable MAPbBr_0.8_I_2.2_ film after it was exposure to three sun illumination by a 532 nm laser beam for 20 min, while the 320 nm film had no change under the same illumination condition. Gaussian fitting was applied to the post‐illumination (200) peak (Figure 4d), from which we can see a small new peak appearing at 2*θ* of 28.5**°**, the (200) peak position for MAPbBr_0.6_I_2.4_—which agrees with the previous study by McGehee's group.^11^ This MAPbBr_0.8_I_2.2_ composition might have the lowest Gibbs free energy, which drives the formation of this phase automatically assisted by photoexcitation. To exclude the possibility that the peak splitting comes from the perovskite decomposition, we kept the illuminated thick films in the dark for two hours, and found the peak splitting can be reversed (blue curve in Figure 4c), which is again consistent to the previous results.^11^ Finally, the light stability of the MAPbBr_0.6_I_2.4_ films with different thickness is also supported by the time integrated PL spectra on the nude perovskite films, as shown in Figure 4e,f. During the five‐minute in situ observation of the thick films illuminated by one sun intensity 532 nm laser, a new PL peak originated from the lower bandgap phase gradually appeared after first 50 sec illumination, while the peak of the PL from the thin film showed no change.

**Figure 4:**
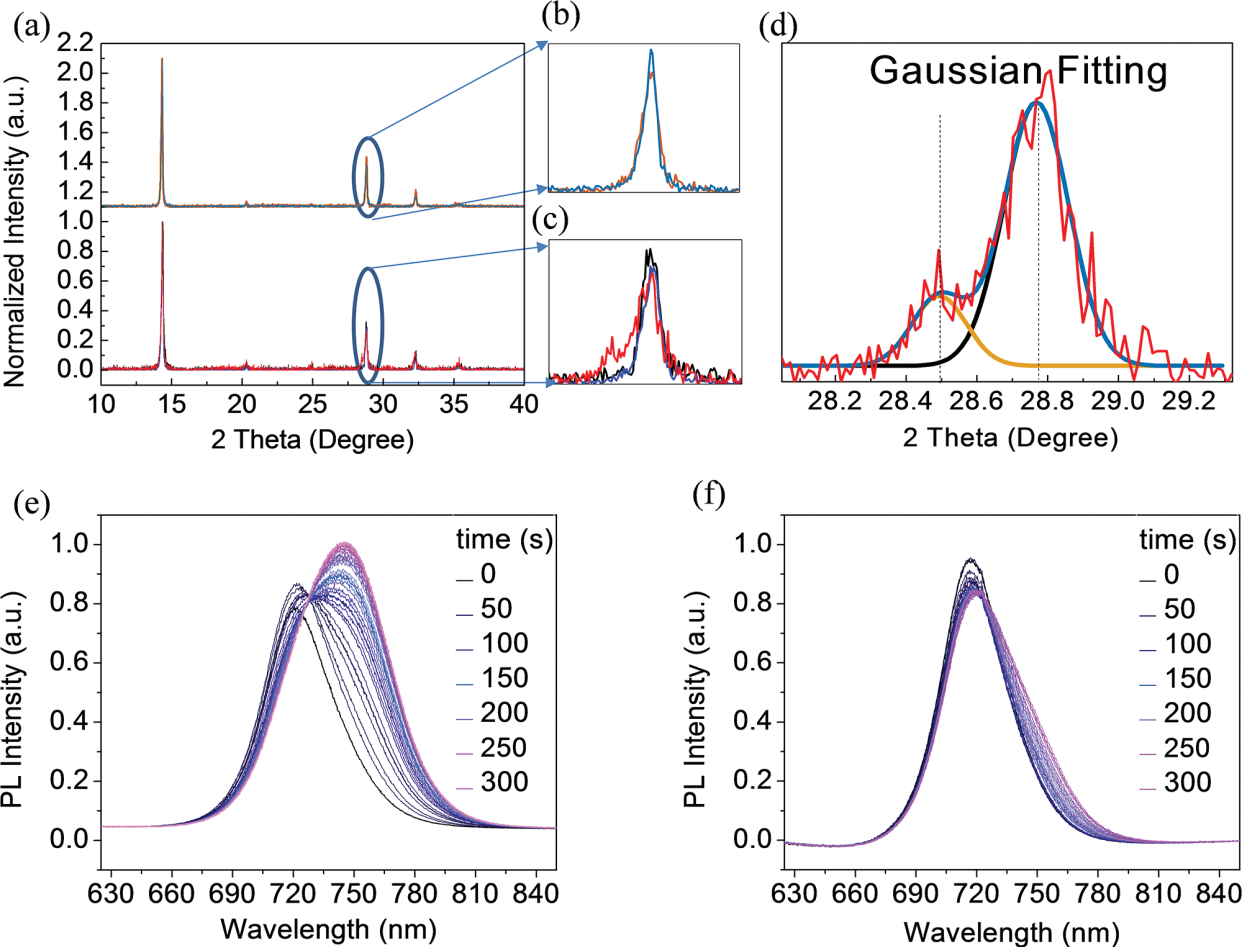
a) XRD pattern for MAPbBr_0.8_I_2.2_ with different perovskite film thickness. b) The (200) XRD peak pre‐ (black) and post‐ (red) illumination for the stable MAPbBr_0.8_I_2.2_ thin film (340 nm). c) The (200) peak before (black) and after (red) exposure to illumination and after recovery (blue) in the dark for 2 hours for the 540 nm MAPbBr_0.8_I_2.2_ film; d) the Gaussian fitting (red) for (200) peak of the 540 nm MAPbBr_0.8_I_2._film after illumination. e–f) PL spectra with an interval of 10 sec for e) the 320 nm MAPbBr_0.8_I_2.2_ film, and f) the 540 nm MAPbBr_0.8_I_2.2_ film, measured during illumination of the films under one‐sun‐intensity 532 nm laser;

In summary, we achieved highly efficient and stable planar heterojunction devices based on the wide bandgap MAPbBr_0.5_I_2.5_. The light stability of the good wider‐bandgap MAPbBr_0.8_I_2.2_ device is demonstrated by a steady photo­current output at maximum power output point over 30 minute under one sun illumination. Microstructure difference between the photo‐stable and photo‐unstable devices is presented by the cross‐section SEM images of the MAPbBr_0.6_I_2.4_ active layer: the spatial homogeneous polycrystalline with large sized grains and the stacking layered polycrystalline with small sized grains, respectively. The PL and EQE spectral change, accompanied with XRD pattern comparison between the MAPbBr_0.8_I_2.2_ thin films with two different microstructures, indicate the enhanced crystallinity and grain size are favorable to retain the homogeneous phase for the mixed halide perovskite during the photo­excitation, thus maintain a stable photocurrent output under the device working condition. The phase instability for mixed halide perovskite was studied with the MAPbBr*_x_*I_3–*x*_ infiltrate into porous TiO_2_ scaffold,^10^ whose microstructure is comparable to the thin film formed on PEDOT:PSS surface or with concentrated precursors in this manuscript. We demonstrate that the microstructure and crystallinity of MAPbBr*_x_*I_3–*x*_ are crucial to achieve the stable wide bandgap perovskite solar cells. This explains why some large bandgap MAPbBr_0.8_I_2.2_ built on mesoporous scaffold device were not stable. Further studies on the role of the grain boundary's area and grain orientation in phase separation of mixed halide perovskite may contribute to a deeper understanding. This work demonstrates the potential of mixed halide perovskite to stay a reliable homogeneous phase in photovoltaic working condition as the wide bandgap light absorber in tandem application.

## Experimental Section

*PbI_2_ and MAI_1_*_–*x*_ *Br_x_ Precursor Preparation*: MAI was synthesized using the method described by Michael M. Lee et al. MABr was synthesized by the reaction of methylamine with a concentrated aqueous solution of hydrobromic acid (23.5 mL, 36.5 wt% in water, Alfa Aesar) at 0 °C for 2 h with constant stirring under nitrogen atmosphere followed by a crystalized, purification and dry process which was the same as the preparation of MAI. MAI_1–*x*_Br*_x_* precursor was prepared by mixing MAI and MABr in 2‐propanol for 1:1.3 weight blend ratio and 62 mg ml^−1^ concentration. PbI_2_ precursor was prepared by dissolving PbI_2_ in DMF with the different concentration 500 mg ml^−1^, 600 mg ml^−1^ and 700 mg ml^−1^.

*Film and Photovoltaic Device Fabrication*: First, hole transport layer was prepared by spin‐coating 0.25 wt% PTAA (toluene) solution doped with 1 wt% F4‐TCNQ at 4000 rpm for 25 sec, and the as‐prepared film thermally annealed at 100 ºC for 10 min. We have already demonstrated that F4‐TCNQ is a suitable dopant to PTAA.^25^ To fabricate 320 nm, 480 nm, and 550 nm thick MAPbI_1–*x*_Br*_x_* films, 500 mg ml^−1^, 600 mg ml^−1^, and 700 mg ml^−1^ PbI_2_ DMF precursor solutions were spun coated on the hole transport layer at 6000 rpm for 35 sec. Then, the as‐prepared PbI_2_ film was dried in a hotplate at 100 °C for 5 min, and followed by spin coating the 62 mg ml^−1^ MAI_1–*x*_Br*_x_* precursor on top of the PbI_2_ layer at 6000 rpm for 35 sec with the subsequent 75 °C 15 min thermal annealing. This process allows the bromide inter‐diffuse into the perovskite structure. Then we increased the annealing temperature to 100 °C, lasting 90 min before cooling down to the room temperature. For photovoltaic devices, 2 wt% solution of PCBM in DCB was spun onto the annealed perovskite film and followed by additional 60‐min annealing. Then the device was completed by sequence depositing 20 nm C_60_, 8 nm BCP and 100 nm Al.

*Device Characterization*: The photocurrent curves were measured under simulated AM 1.5G irradiation (100 mW cm^−2^) using a Xenon‐lamp‐based solar simulator (Oriel 67005, 150 W Solar Simulator). A Schott visible‐color glass‐filtered (KG5 color‐filtered) Si diode (Hamamatsu S1133) was used to calibrate the light intensity before photocurrent measurement. The device area was 7.5 mm^2^. EQE was measured with a Newport QE measurement kit by focusing a monochromatic beam of light onto the devices.

*Film Characterization*: XRD measurements were performed with the PANalytical Empyrean Diffractometer with Bragg–Brentano parafocusing geometry, the 3 kW Cu Kα source, and the PIXcel 3D detector. The scan rate is 0.02 s per step and 0.5 s per step with an angular range of 10–60. The single path absorption was measured using an Evolution 201 UV–visible spectrometer (Thermo Scientific) with the scan rate 1 nm per step and 0.5 s per step in the range of 350–900 nm. The PL spectrum was measured by iHR320 Photoluminescence Spectroscopy at room temperature. A 532 nm green laser with an intensity of 100 mW cm^−2^ from Laserglow Technologies was used as excitation source in PL measurement. PL integration time is 5 secs and the slit is 0.12 mm. PL intensity was collected every 10 sec continuously for 30 min. The cross‐section SEM images were taken from the Quanta 200 FEG Environmental Scanning Electron Microscope (ESEM) using a field‐emission gun (FEG) electron source to scan the gold coated cross‐section area morphology.
